# Supplementary material for: Sexual dimorphism of the human fetal pelvis exists at the onset of primary ossification
Source: Commun Biol. 2024 May 7;7:538. doi: 10.1038/s42003-024-06156-y (PMC11076513; doi:10.1038/s42003-024-06156-y)
Supplement: Supplementary file 2 — Supplementary information [file 42003_2024_6156_MOESM2_ESM.pdf]

Supplementary Information

Article Title

Sexual dimorphism of the human fetal pelvis exists at the onset of primary ossification

Authors

Toru Kanahashi[1], Jun Matsubayashi[2], Hirohiko Imai[3], Shigehito Yamada[1,4], Hiroki Otani[5], Tetsuya Takakuwa[1]

[1]Human Health Science, Graduate School of Medicine, Kyoto University, Kyoto, Japan

[2]Center for Clinical Research and Advanced Medicine, Shiga University of Medical Science, Shiga, Japan

[3]Department of Systems Science, Graduate School of Informatics, Kyoto University, Kyoto, Japan

[4]Congenital Anomaly Research Center, Graduate School of Medicine, Kyoto University, Kyoto, Japan

[5]Department of Developmental Biology, Faculty of Medicine, Shimane University, Shimane, Japan

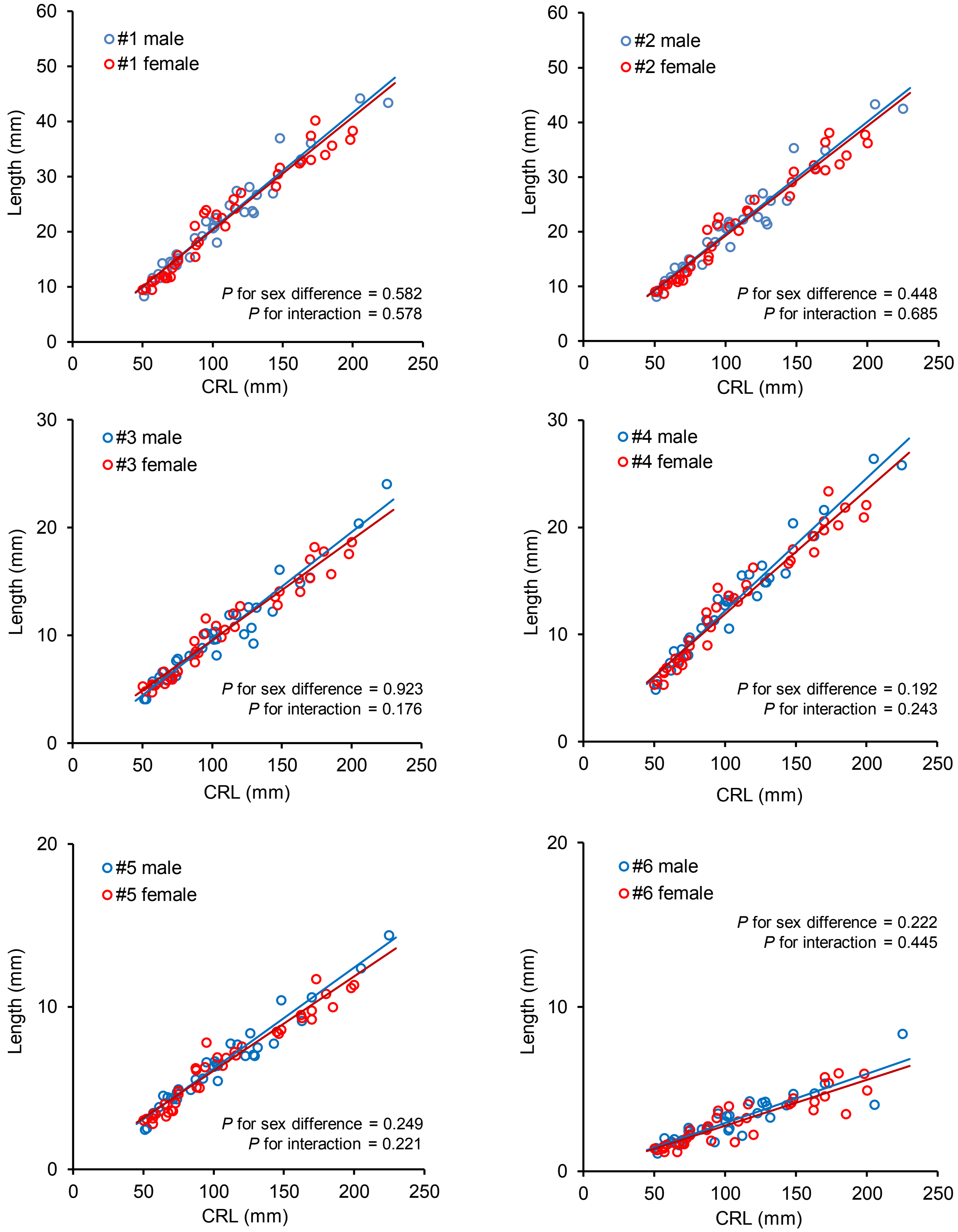

Supplementary Figure 1. Pelvimetry of the greater pelvis (a part). The letters correspond with the dimensions described in Figure 2 and Table 1. The male and female regression lines are represented in blue and red, respectively. CRL, crown-rump length.

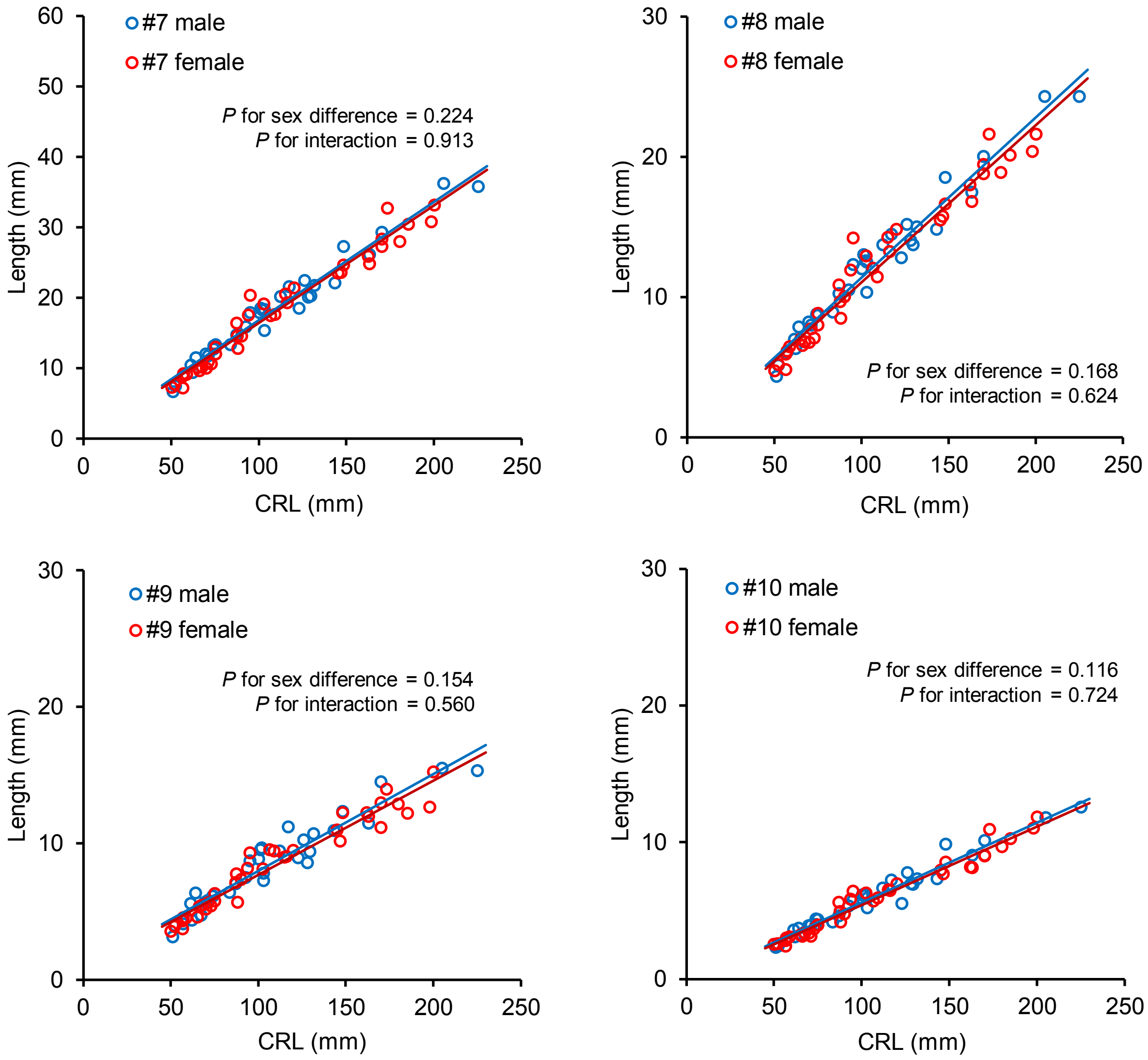

Supplementary Figure 2. Pelvimetry of the greater pelvis (a part). The letters correspond with the dimensions described in Figure 2 and Table 1. The male and female regression lines are represented in blue and red, respectively. CRL, crown-rump length.

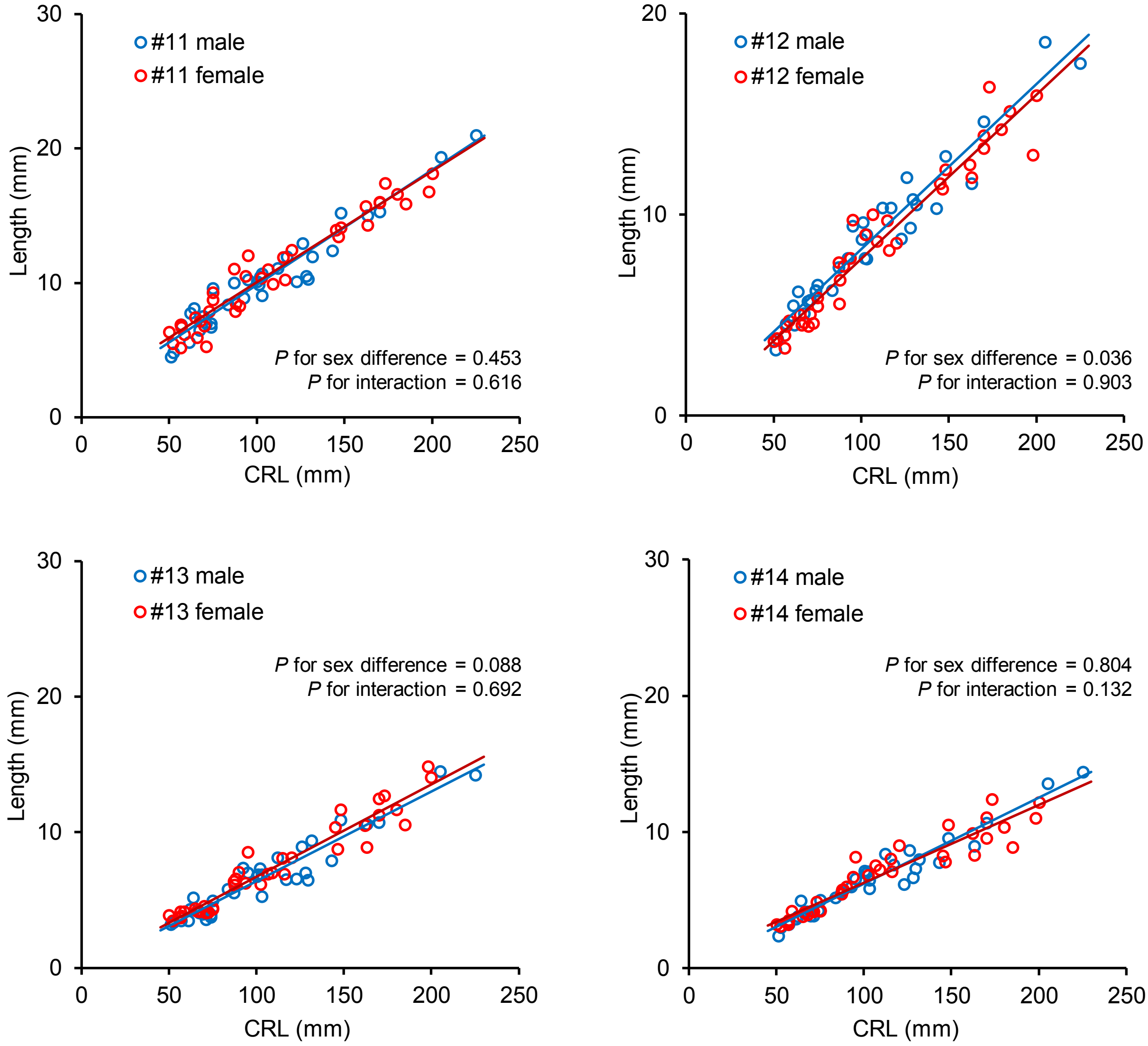

Supplementary Figure 3. Pelvimetry of the lesser pelvis (a part). The letters correspond with the dimensions described in Figure 2 and Table 1. The male and female regression lines are represented in blue and red, respectively. CRL, crown-rump length.

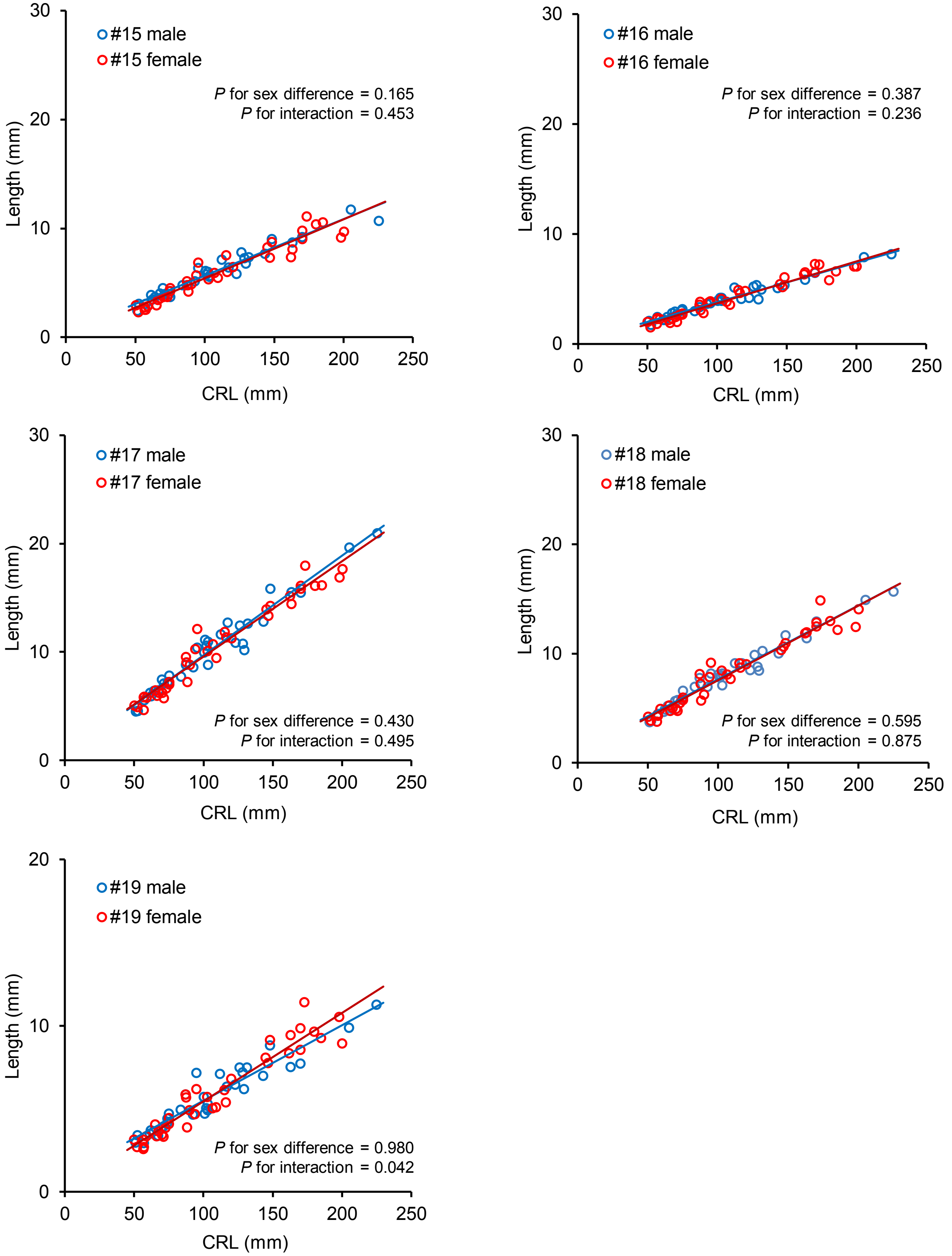

Supplementary Figure 4. Pelvimetry of the lesser pelvis (a part). The letters correspond with the dimensions described in Figure 2 and Table 1. The male and female regression lines are represented in blue and red, respectively. CRL, crown-rump length.

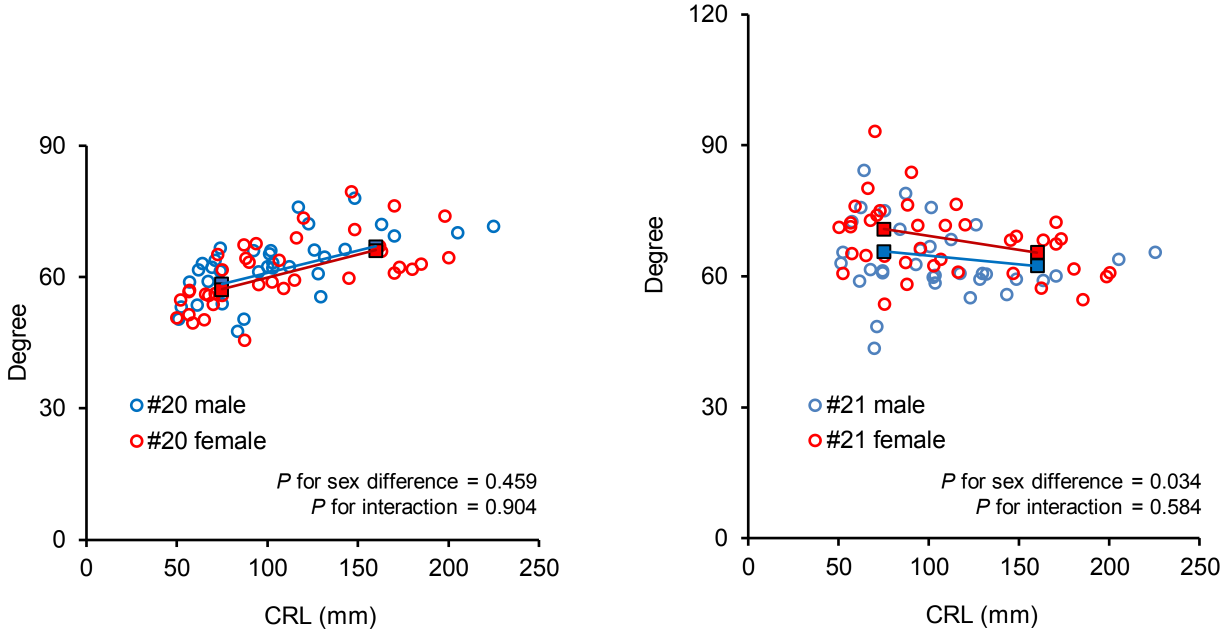

Supplementary Figure 5. Iliac crest angle and subpubic angle. The letters correspond with the dimensions described in Figure 2 and Table 1. Blue and red squares indicate the estimated means for males and females in each CRL subgroup, respectively. CRL, crown-rump length.

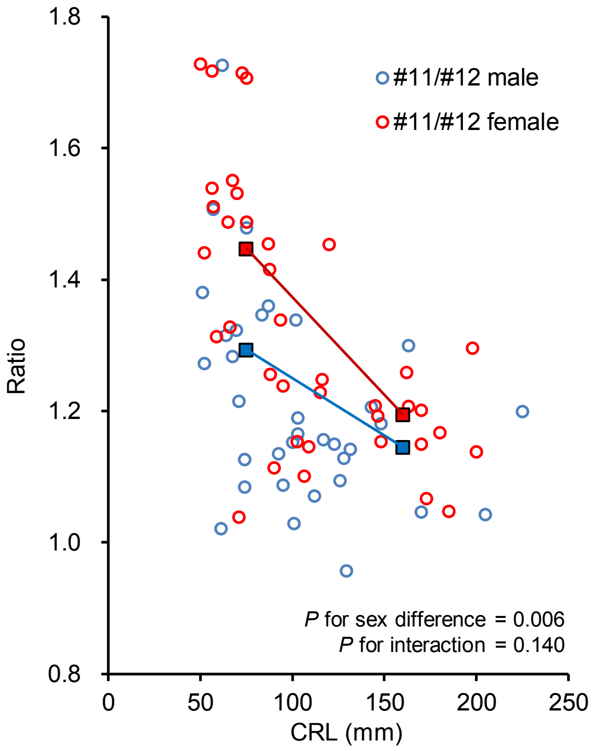

Supplementary Figure 6. Ratio of the pelvic inlet. The letters correspond with the dimensions described in Figure 2 and Table 1. Blue and red squares indicate the estimated means for males and females in each CRL subgroup, respectively. CRL, crown-rump length.

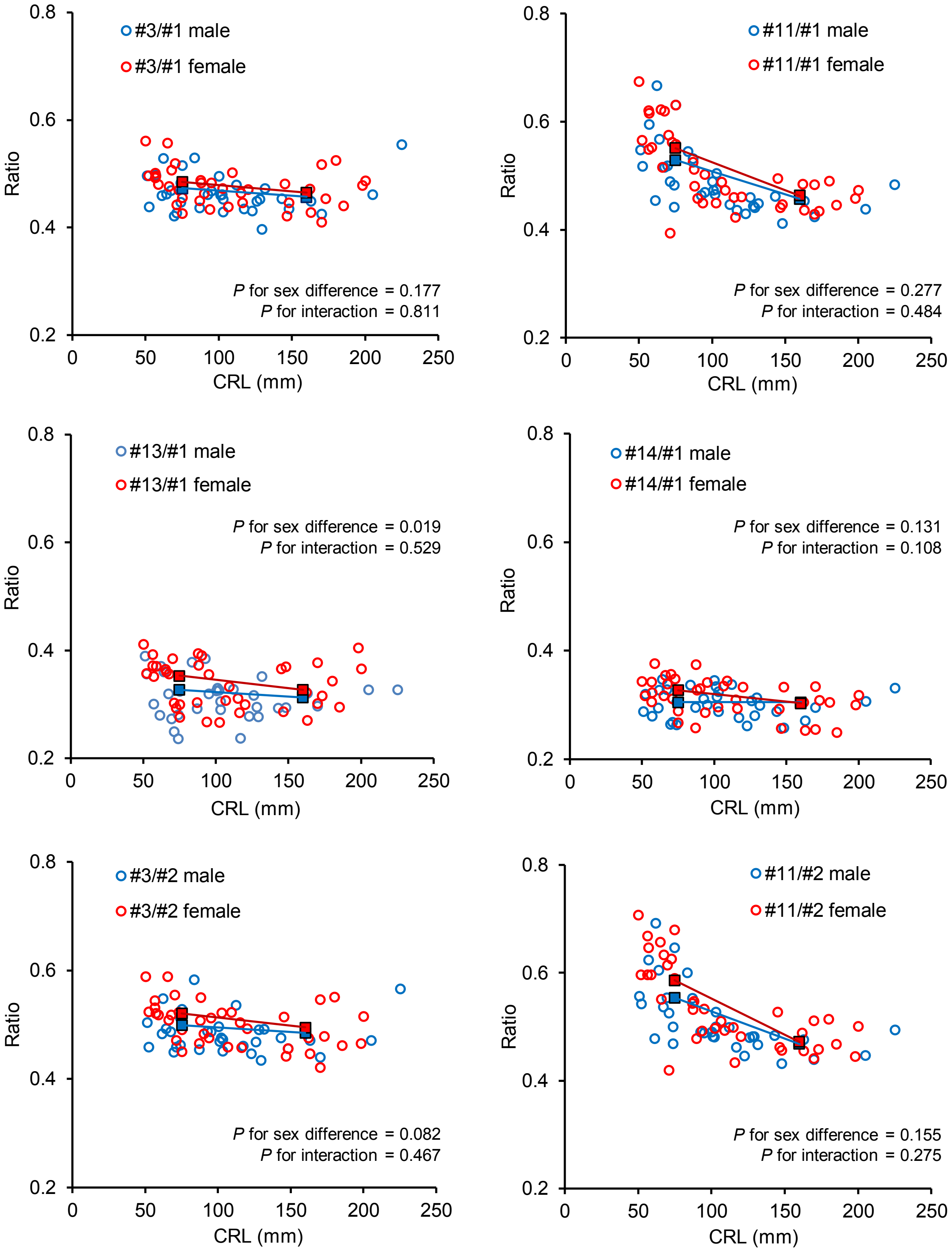

Supplementary Figure 7. Ratio of the transverse diameters of the pelvis (a part). The letters correspond with the dimensions described in Figure 2 and Table 1. Blue and red squares indicate the estimated means for males and females in each CRL subgroup, respectively. CRL, crown-rump length.

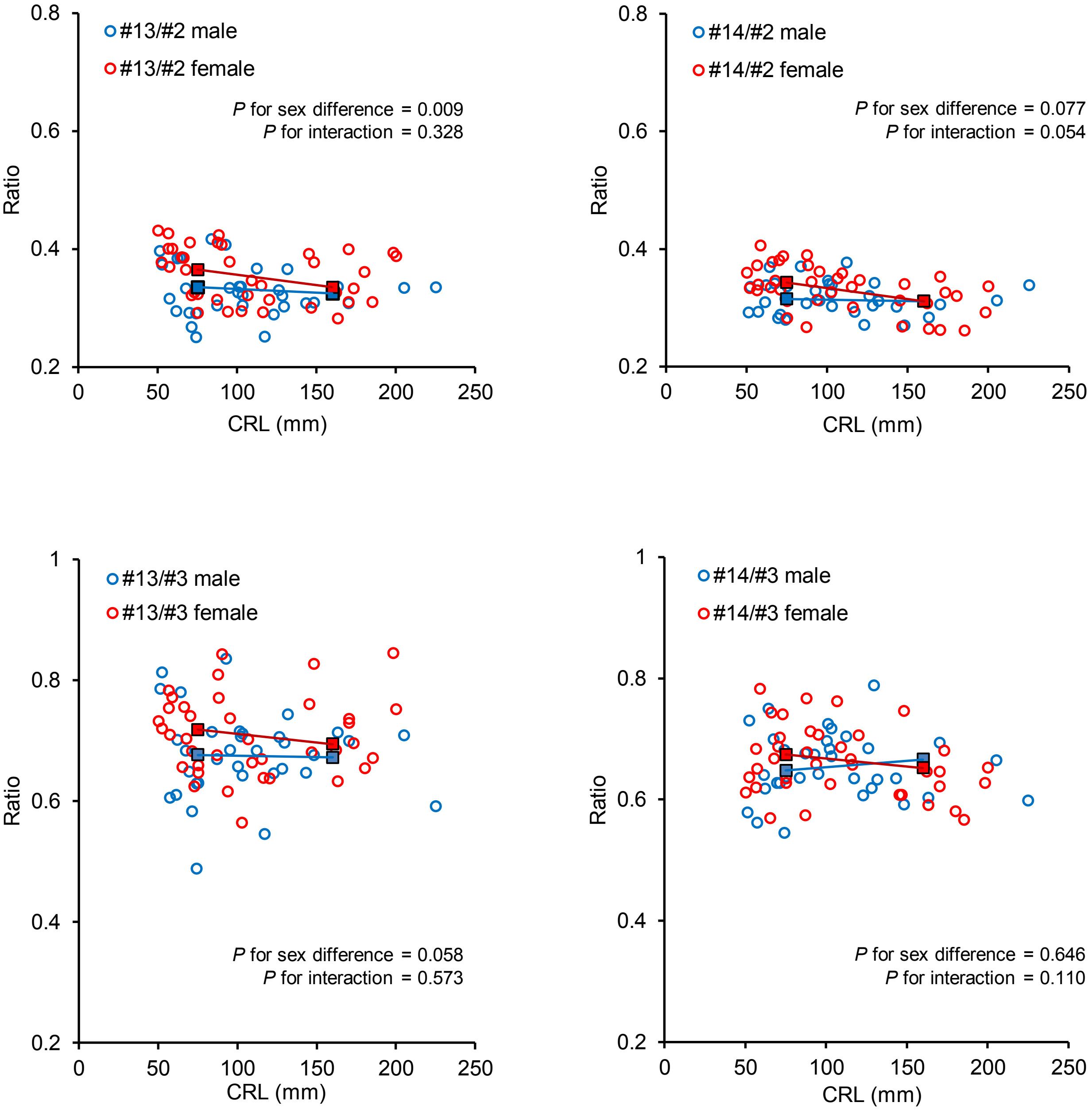

Supplementary Figure 8. Ratio of the transverse diameters of the pelvis (a part). The letters correspond with the dimensions described in Figure 2 and Table 1. Blue and red squares indicate the estimated means for males and females in each CRL subgroup, respectively. CRL, crown-rump length.

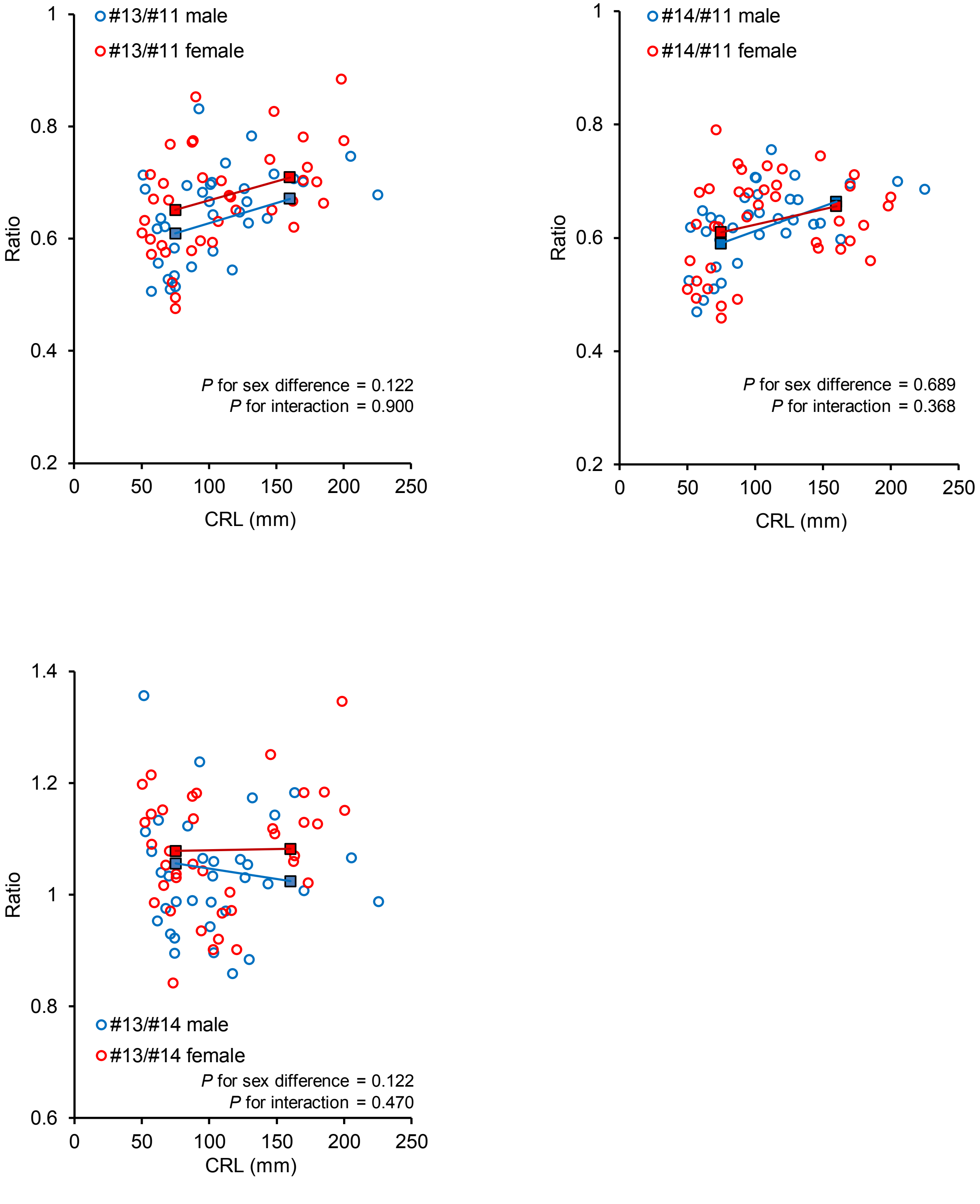

Supplementary Figure 9. Ratio of the transverse diameters of the pelvis. The letters correspond with the dimensions described in Figure 2 and Table 1. Blue and red squares indicate the estimated means for males and females in each CRL subgroup, respectively. CRL, crown-rump length.

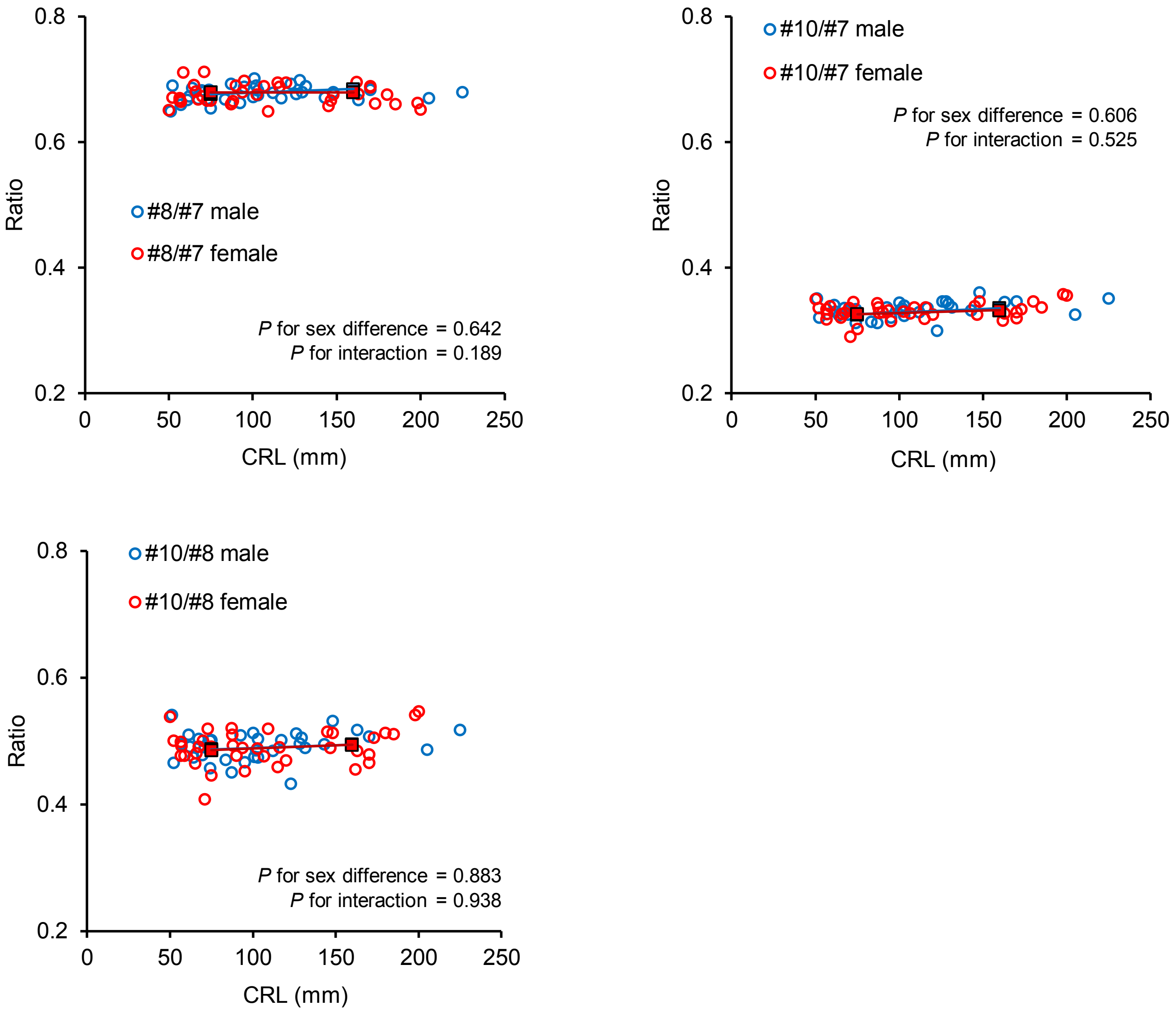

Supplementary Figure 10. Ratio of the cranial-caudal diameters of the pelvis. The letters correspond with the dimensions described in Figure 2 and Table 1. Blue and red squares indicate the estimated means for males and females in each CRL subgroup, respectively. CRL, crown-rump length.

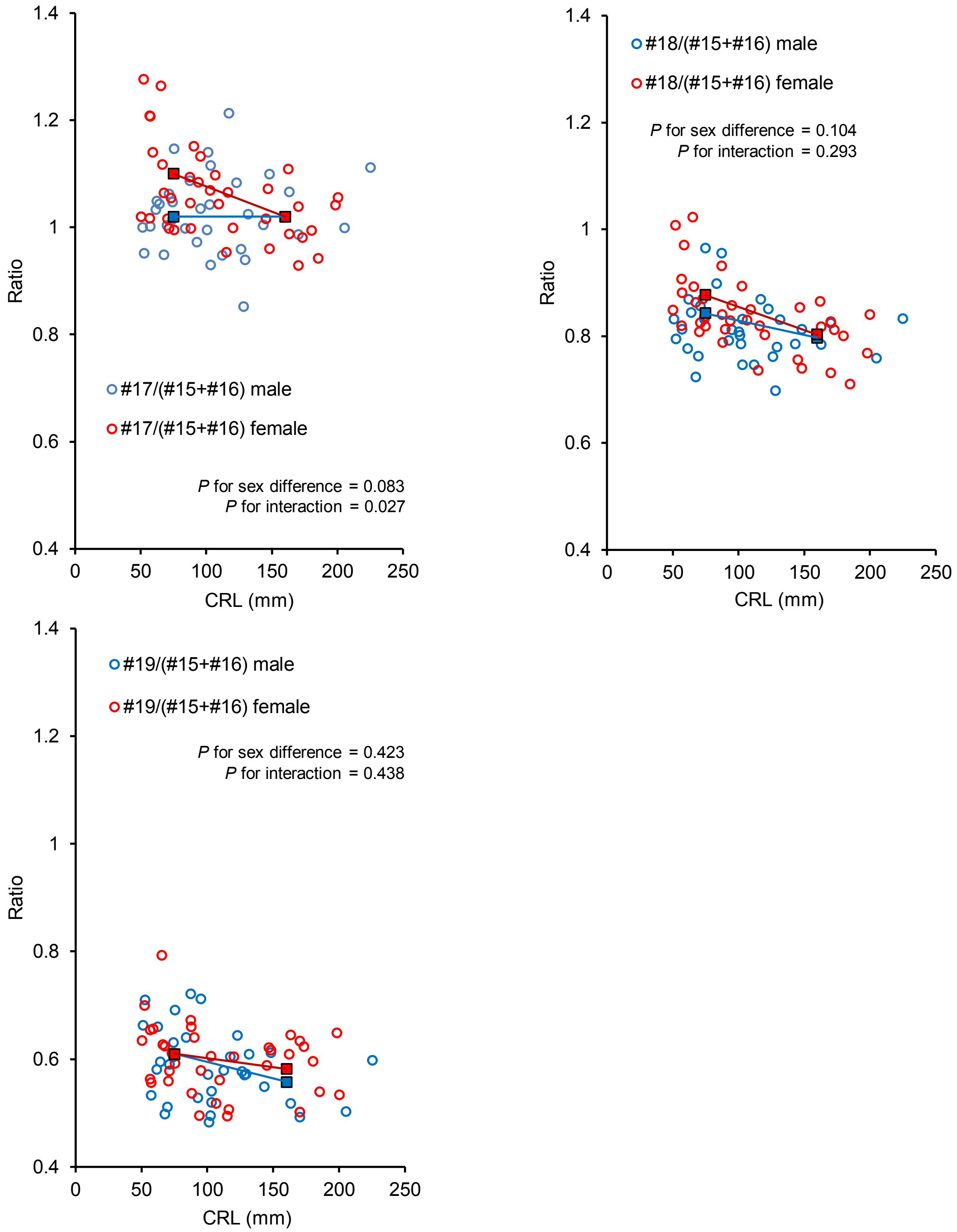

Supplementary Figure 11. Aspect ratios length-to-width of the sacrum. The letters correspond with the dimensions described in Figure 2 and Table 1. Blue and red squares indicate the estimated means for males and females in each CRL subgroup, respectively. CRL, crown-rump length.

Supplementary Table 1. Sex differences in linear pelvic dimensions using multiple regression analysis

Measurement	Sex difference				Effect of CRL		Interaction
	Male (M), LS mean*, mm [95% CI]	Female (F), LS mean*, mm [95% CI]	Difference (F – M), LS mean*, mm [95% CI]	*P* value	Coefficient (per 10-mm increase) [95% CI]	*P* value	*P* value
**Linear dimensions of the greater pelvis**							
#1	22.0 [21.3, 22.7]	21.7 [21.0, 22.4]	−0.3 [−1.3, 0.7]	0.582	2.1 [2.0, 2.2]	<0.001	0.578
#2	21.0 [20.3, 21.7]	20.7 [20.0, 21.3]	−0.4 [−1.3, 0.6]	0.448	2.0 [1.9, 2.1]	<0.001	0.685
#3	10.2 [9.8, 10.5]	10.2 [9.9, 10.5]	0.0 [−0.5,0.5]	0.923	1.0 [0.9, 1.0]	<0.001	0.176
#4	13.1 [12.7, 13.5]	12.7 [12.3, 13.1]	−0.4 [−0.9, 0.2]	0.192	1.2 [1.1, 1.3]	<0.001	0.243
#5	6.6 [6.4, 6.8]	6.4 [6.2, 6.6]	−0.2 [−0.5, 0.1]	0.249	0.6 [0.6, 0.6]	<0.001	0.221
#6	3.2 [3.0, 3.4]	3.0 [2.8, 3.2]	−0.2 [−0.5, 0.1]	0.222	0.3 [0.3, 0.3]	<0.001	0.445
#7	18.0 [17.5, 18.5]	17.6 [17.1, 18.1]	−0.4 [−1.1, 0.3]	0.224	1.7 [1.6, 1.8]	<0.001	0.913
#8	12.2 [11.9, 12.6]	11.9 [11.6, 12.2]	−0.3 [−0.8, 0.1]	0.168	1.1 [1.1, 1.2]	<0.001	0.624
#9	8.5 [8.2, 8.8]	8.2 [7.9, 8.5]	−0.3 [−0.7, 0.1]	0.154	0.7 [0.7, 0.8]	<0.001	0.560
#10	6.0 [5.9, 6.2]	5.9 [5.7, 6.0]	−0.2 [−0.4, 0.1]	0.116	0.6 [0.6, 0.6]	<0.001	0.724
**Linear dimensions of the lesser pelvis**							
#11	10.4 [10.1, 10.7]	10.6 [10.3, 10.9]	0.2 [−0.3, 0.6]	0.453	0.8 [0.8, 0.9]	<0.001	0.616
#12	8.8 [8.5, 9.2]	8.4 [8.1, 8.7]	−0.5 [−0.9, 0.0]	0.036	0.8 [0.8, 0.9]	<0.001	0.903
#13	6.9 [6.5, 7.2]	7.2 [6.9, 7.5]	0.4 [−0.1, 0.8]	0.088	0.7 [0.6, 0.7]	<0.001	0.692
#14	6.6 [6.4, 6.9]	6.7 [6.4, 7.0]	0.0 [−0.3, 0.4]	0.804	0.6 [0.6, 0.6]	<0.001	0.132

Linear dimensions of the sacrum							
#15	6.0 [5.8, 6.2]	5.8 [5.6, 6.0]	−0.2 [−0.5, 0.1]	0.165	0.5 [0.5, 0.6]	<0.001	0.453
#16	4.1 [4.0, 4.2]	4.0 [3.9, 4.1]	−0.1 [−0.3, 0.1]	0.387	0.4 [0.4, 0.4]	<0.001	0.236
#17	10.4 [10.1, 10.7]	10.2 [9.9, 10.5]	−0.2 [−0.6, 0.2]	0.430	0.9 [0.9, 0.9]	<0.001	0.495
#18	8.1 [7.9, 8.4]	8.1 [7.8, 8.3]	−0.1 [−0.4, 0.2]	0.595	0.7 [0.6, 0.7]	<0.001	0.875
#19	5.8 [5.6, 6.0]	5.8 [5.6, 6.0]	0.0 [−0.3, 0.3]	0.980	0.5 [0.5, 0.5]	<0.001	0.042

* LS mean is the estimated mean of the pelvimetric values if the CRL has a mean value. *CI,* confidence interval; *CRL*, crown-rump length; *LS,* least squares.

Supplementary Table 2. Sex differences in pelvic angles using multiple regression analysis

Measurement	Sex difference				Subgroup based on CRL†		Interaction
	Male (M),<br>LS mean*, degree<br>[95% CI]	Female (F),<br>LS mean*, degree<br>[95% CI]	Difference (F – M),<br>LS mean*, degree<br>[95% CI]	*P* value	Difference (large – small),<br>LS mean§, degree<br>[95% CI]	*P* value	*P* value
#20	62.7 [60.6, 64.8]	61.6 [59.6, 63.6]	−1.1 [−4.0, 1.8]	0.459	8.7 [5.8, 11.6]	<0.001	0.904
#21	64.0 [61.3, 66.8]	68.1 [65.5, 70.7]	4.1 [0.3, 7.9]	0.034	−4.3 [−8.1, −0.5]	0.026	0.584

* LS mean is the estimated mean of the measured value if both sex groups have a 1:1 ratio of subgroups based on the CRL. † Subgroups are defined as two groups with CRLs <100 mm (small group) and ≥100 mm (large group). § LS mean is the estimated mean of the measured value if both subgroups based on the CRL have the same sex ratio. *CI,* confidence interval; *CRL*, crown-rump length; *LS,* least squares.

Supplementary Table 3. Sex differences in pelvic ratios using multiple regression analysis

Measurement	Sex difference				Subgroup based on CRL†		Interaction
	Male (M), LS mean* [95% CI]	Female (F), LS mean* [95% CI]	Difference (F – M), LS mean* [95% CI]	*P* value	Difference (large – small), LS mean§ [95% CI]	*P* value	*P* value
**Ratio of the pelvic inlet**							
$\frac{\#11}{\#12}$	1.22 [1.17, 1.27]	1.32 [1.27, 1.37]	0.10 [0.03, 0.17]	0.006	−0.20 [−0.27, −0.13]	<0.001	0.140
**Ratios of the transverse diameters of the pelvis**							
$\frac{\#3}{\#1}$	0.46 [0.45, 0.48]	0.47 [0.46, 0.49]	0.01 [−0.01, 0.03]	0.177	−0.02 [−0.03, 0.00]	0.028	0.811
$\frac{\#11}{\#1}$	0.49 [0.47, 0.51]	0.50 [0.49, 0.52]	0.01 [−0.01, 0.04]	0.277	−0.08 [−0.11, −0.06]	<0.001	0.484
$\frac{\#13}{\#1}$	0.31 [0.30, 0.33]	0.34 [0.32, 0.35]	0.02 [0.00, 0.04]	0.019	−0.02 [−0.04, 0.00]	0.047	0.529
$\frac{\#14}{\#1}$	0.30 [0.29, 0.31]	0.31 [0.30, 0.32]	0.01 [0.00, 0.02]	0.131	−0.01 [−0.03, 0.00]	0.066	0.108
$\frac{\#3}{\#2}$	0.49 [0.47, 0.50]	0.50 [0.49 0.51]	0.02 [0.00, 0.03]	0.082	−0.03 [−0.04, −0.01]	0.005	0.467
$\frac{\#11}{\#2}$	0.52 [0.50, 0.53]	0.53 [0.52, 0.55]	0.02 [−0.01 0.04]	0.155	−0.09 [−0.12, −0.07]	<0.001	0.275
$\frac{\#13}{\#2}$	0.33 [0.31, 0.34]	0.36 [0.34, 0.37]	0.03 [0.01, 0.05]	0.009	−0.02 [−0.04, −0.01]	0.014	0.328
$\frac{\#14}{\#2}$	0.32 [0.31, 0.33]	0.33 [0.32, 0.34]	0.01 [0.00, 0.03]	0.077	−0.02 [−0.03, 0.00]	0.022	0.054

$\frac{\#13}{\#3}$	0.68 [0.65, 0.70]	0.71 [0.69, 0.73]	0.03 [0.00, 0.06]	0.058	−0.01 [−0.05, 0.02]	0.414	0.573
$\frac{\#14}{\#3}$	0.65 [0.64, 0.67]	0.66 [0.64, 0.68]	0.01 [−0.02, 0.03]	0.646	0.00 [−0.03, 0.02]	0.837	0.110
$\frac{\#13}{\#11}$	0.64 [0.61, 0.67]	0.67 [0.65, 0.70]	0.03 [−0.01, 0.07]	0.122	0.06 [0.02, 0.10]	0.002	0.900
$\frac{\#14}{\#11}$	0.62 [0.60, 0.65]	0.63 [0.61, 0.65]	0.01 [−0.03, 0.04]	0.689	0.07 [0.04, 0.10]	<0.001	0.368
$\frac{\#13}{\#14}$	1.04 [1.00, 1.07]	1.08 [1.04, 1.11]	0.04 [−0.01, 0.09]	0.122	−0.01 [−0.06, 0.04]	0.600	0.470
**Ratios of the cranial-to-caudal diameters of the pelvis**							
$\frac{\#8}{\#7}$	0.68 [0.67, 0.68]	0.68 [0.67, 0.68]	0.00 [−0.01, 0.01]	0.642	0.00 [0.00, 0.01]	0.335	0.189
$\frac{\#10}{\#7}$	0.33 [0.33, 0.34]	0.33 [0.33, 0.34]	0.00 [−0.01, 0.00]	0.606	0.01 [0.00, 0.01]	0.014	0.525
$\frac{\#10}{\#8}$	0.49 [0.48, 0.50]	0.49 [0.48, 0.50]	0.00 [−0.01, 0.01]	0.883	0.01 [0.00, 0.02]	0.156	0.938
**Aspect ratios length-to-width of the sacrum**							
$\frac{\#17}{\#15 + \#16}$	1.03 [1.00, 1.05]	1.06 [1.03, 1.08]	0.03 [0.00, 0.07]	0.083	−0.04 [−0.07, 0.00]	0.045	0.027
$\frac{\#18}{\#15 + \#16}$	0.82 [0.80, 0.83]	0.84 [0.82, 0.86]	0.02 [−0.01, 0.05]	0.104	−0.05 [−0.08, −0.03]	<0.001	0.293
$\frac{\#19}{\#15 + \#16}$	0.59 [0.57, 0.61]	0.60 [0.58, 0.62]	0.01 [−0.02, 0.04]	0.423	−0.05 [−0.08, −0.02]	0.001	0.438

* LS mean is the estimated mean of the measured value if both sex groups have a 1:1 ratio of subgroups based on the CRL. † Subgroups are defined as two groups with CRLs <100 mm (small group) and ≥100 mm (large group). § LS mean is the estimated mean of the measured value if both subgroups based on

the CRL have the same sex ratio. *CI,* confidence interval; *CRL*, crown-rump length; *LS,* least squares.

Supplementary Table 4. Multiple regression analysis for the subpubic angle (#21) and the three ratios (#11/#12, #13/#1, and #13/#2) with CRL as a continuous variable

**Measurement**	**Sex difference**				**Effect of CRL**		**Interaction**
	**Male (M), LS mean* [95% CI]**	**Female (F), LS mean* [95% CI]**	**Difference (F – M), LS mean* [95% CI]**	***P* value**	**Coefficient (per 10-mm increase) [95% CI]**	***P* value**	***P* value**
#21	63.8 [61.1, 66.6]	68.4 [65.8, 71.0]	4.5 [0.8, 8.3]	0.019	−0.5 [−0.9, −0.1]	0.025	0.449
$\frac{\#11}{\#12}$	1.21 [1.16, 1.26]	1.33 [1.28, 1.38]	0.12 [0.05, 0.19]	0.001	−0.02 [-0.03, −0.01]	<0.001	0148
$\frac{\#13}{\#1}$	0.31 [0.30, 0.33]	0.34 [0.32, 0.35]	0.02 [0.00, 0.04]	0.015	0.00 [0.00, 0.00]	0.286	0.779
$\frac{\#13}{\#2}$	0.33 [0.31, 0.34]	0.36 [0.34, 0.37]	0.03 [0.01, 0.05]	0.006	0.00 [0.00, 0.00]	0.110	0.546

Note. A multiple regression model was used in which the independent variables were sex, CRL (as a continuous variable), and the interaction between sex and CRL. The unit of #21 is a degree. * LS mean is the estimated mean of the pelvimetric values if the CRL has a mean value. *CI,* confidence interval; *CRL*, crown-rump length; *LS,* least squares.

Supplementary Table 5. Polynomial multiple regression analysis for the subpubic angle (#21) and three ratios (#11/#12, #13/#1, and #13/#2) with a squared CRL term

Measurement	Sex difference				CRL	Squared CRL
	**Male (M), LS mean* [95% CI]**	**Female (F), LS mean* [95% CI]**	**Difference (F – M), LS mean* [95% CI]**	***P* value**	***P* value**	***P* value**
#21	63.5 [60.3, 66.8]	68.0 [64.8, 71.3]	4.5 [0.7, 8.3]	0.020	0.034	0.746
$\frac{\#11}{\#12}$	1.16 [1.10, 1.21]	1.27 [1.21, 1.33]	0.12 [0.05, 0.18]	0.001	<0.001	0.001
$\frac{\#13}{\#1}$	0.30 [0.28, 0.32]	0.32 [0.31, 0.34]	0.02 [0.00, 0.04]	0.015	0.007	0.003
$\frac{\#13}{\#2}$	0.32 [0.30, 0.33]	0.34 [0.33, 0.36]	0.03 [0.01, 0.05]	0.006	0.003	0.008

Note. A polynomial multiple regression model was used in which the independent variables were sex, CRL (as a continuous variable), and squared CRL. The unit of #21 is a degree. * LS mean is the estimated mean of the pelvimetric values if the CRL has a mean value. *CI,* confidence interval; *CRL*, crown-rump length; *LS,* least squares.

Supplementary Table 6. Samples used in this study

ID	sample location	CRL (mm)	sex	MRI
F2580	Kyoto	50	F	7T
51262	Kyoto	51	M	7T
51128	Kyoto	52	F	7T
34365	Kyoto	52	M	7T
20799	Kyoto	57	F	7T
52201	Kyoto	57	F	7T
36113	Kyoto	57	F	7T
F3049	Kyoto	57	M	7T
33087	Shimane	59	F	7T
F1728	Kyoto	61	M	7T
51272	Kyoto	62	M	7T
F2214-2	Kyoto	64	M	7T
F2148	Kyoto	65	F	7T
36175	Kyoto	66	F	7T
71030	Shimane	67	M	7T
35922	Kyoto	68	F	7T
F2133	Shimane	70	M	7T
37334	Kyoto	70	F	7T
52248	Kyoto	71	F	7T
F2373	Shimane	71	M	7T
36530	Kyoto	73	F	7T
52770	Kyoto	74	M	7T
F2149	Shimane	74	M	7T
51732	Kyoto	75	F	7T
52559	Kyoto	75	M	7T
F1088	Kyoto	75	F	7T
38641	Kyoto	84	M	7T
34192	Kyoto	87	F	7T
50673	Kyoto	87	M	7T
37304	Shimane	88	F	7T
F2286	Shimane	88	F	h-7T
53514	Kyoto	90	F	3T

F2230	Shimane	93	M	h-7T
F15	Shimane	94	F	h-7T
37729	Shimane	95	F	h-7T
F1874	Shimane	95	M	h-7T
37827	Shimane	100	M	h-7T
F1879	Shimane	101	M	h-7T
F1780	Shimane	102	M	h-7T
F2070	Shimane	103	F	h-7T
70323	Shimane	103	M	h-7T
F2245	Shimane	103	M	h-7T
F2139	Shimane	107	F	h-7T
F1925	Shimane	109	F	h-7T
F1915	Shimane	112	M	h-7T
F2011	Shimane	115	F	h-7T
53591	Kyoto	116	F	3T
F1892	Shimane	117	M	h-7T
F2275	Shimane	120	F	h-7T
53273	Kyoto	123	M	3T
F2225	Shimane	126	M	h-7T
37626	Shimane	128	M	h-7T
37866	Shimane	129	M	h-7T
53605	Kyoto	132	M	3T
53468	Kyoto	143	M	3T
53598	Kyoto	145	F	3T
53469	Kyoto	147	F	3T
52038	Kyoto	148	M	3T
91517	Kyoto	148	F	3T
53512	Kyoto	162	F	3T
53444	Kyoto	163	F	3T
53471	Kyoto	163	M	3T
53485	Kyoto	170	F	3T
53503	Kyoto	170	M	3T
53590	Kyoto	170	F	3T
91473	Kyoto	173	F	3T
53516	Kyoto	180	F	3T

53467	Kyoto	185	F	3T
53513	Kyoto	198	F	3T
53521	Kyoto	200	F	3T
53588	Kyoto	205	M	3T
53570	Kyoto	225	M	3T

CRL, crown-rump length; *Kyoto*, Congenital Anomaly Research Center of Kyoto University; *Shimane*, Shimane University; *F*, female; *M*, male; *7T*, BioSpec 70/20 USR Bruker BioSpin MRI GmbH Ettlingen Germany; *3T*, Magnetom Prisma Siemens Healthineers Erlangen Germany; *h-7T*, Magnetom 7T Siemens Healthineers Erlangen Germany. This MRI was intended for use in humans.
