# Supplementary material for: Sexual dimorphism of the human fetal pelvis exists at the onset of primary ossification
Source: Commun Biol. 2024 May 7;7:538. doi: 10.1038/s42003-024-06156-y (PMC11076513; doi:10.1038/s42003-024-06156-y)
Supplement: Supplementary file 3 — Description of Additional Supplementary Files [file 42003_2024_6156_MOESM3_ESM.pdf]

# Description of Additional Supplementary Files

**File name:** Supplementary Movie 1

**Description:** Reconstructed image in a male fetus (crown-rump length, 70 mm). The light blue areas indicate the areas of primary ossification. ASIS, anterior superior iliac spine; IS, ischial spine.

**File name:** Supplementary Movie 2

**Descrip ion:** Reconstructed image in a female fetus (crown-rump length, 70 mm). The light blue areas indicate the areas of primary ossificafion. ASIS, anterior superior iliac spine; IS, ischial spine.

**File name:** Supplementary Movie 3

**Description:** Reconstructed image in a male fetus (crown-rump length, 70 mm). The light blue areas indicate the areas of primary ossification. ASIS, anterior superior iliac spine; IS, ischial spine; IT, ischial tuberosity; PS, pubic symphysis.

**File name:** Supplementary Movie 4

**Description:** Reconstructed image in a female fetus (crown-rump length, 70 mm). The light blue areas indicate the areas of primary ossification. ASIS, anterior superior iliac spine; IS, ischial spine; IT, ischial tuberosity; PS, pubic symphysis.

**File name:** Supplementary Data 1

**Description:** Source data of all measurements of pelvimetry.
