# Supplementary material for: Sexual dimorphism of the human fetal pelvis exists at the onset of primary ossification
Source: Commun Biol. 2024 May 7;7:538. doi: 10.1038/s42003-024-06156-y (PMC11076513; doi:10.1038/s42003-024-06156-y)
Supplement: Supplementary file 9 — Reporting Summary [file 42003_2024_6156_MOESM9_ESM.pdf]

nature portfolio

Corresponding author(s): Toru Kanahashi

Last updated by author(s): Apr 1, 2024

Reporting Summary

Nature Portfolio wishes to improve the reproducibility of the work that we publish. This form provides structure for consistency and transparency in reporting. For further information on Nature Portfolio policies, see our Editorial Policies and the Editorial Policy Checklist.

Statistics

For all statistical analyses, confirm that the following items are present in the figure legend, table legend, main text, or Methods section.

n/a	Confirmed	
☐	☒	The exact sample size (*n*) for each experimental group/condition, given as a discrete number and unit of measurement
☐	☒	A statement on whether measurements were taken from distinct samples or whether the same sample was measured repeatedly
☐	☒	The statistical test(s) used AND whether they are one- or two-sided<br>*Only common tests should be described solely by name; describe more complex techniques in the Methods section.*
☐	☒	A description of all covariates tested
☐	☒	A description of any assumptions or corrections, such as tests of normality and adjustment for multiple comparisons
☐	☒	A full description of the statistical parameters including central tendency (e.g. means) or other basic estimates (e.g. regression coefficient) AND variation (e.g. standard deviation) or associated estimates of uncertainty (e.g. confidence intervals)
☐	☒	For null hypothesis testing, the test statistic (e.g. *F*, *t*, *r*) with confidence intervals, effect sizes, degrees of freedom and *P* value noted<br>*Give P values as exact values whenever suitable.*
☒	☐	For Bayesian analysis, information on the choice of priors and Markov chain Monte Carlo settings
☒	☐	For hierarchical and complex designs, identification of the appropriate level for tests and full reporting of outcomes
☐	☒	Estimates of effect sizes (e.g. Cohen's *d*, Pearson's *r*), indicating how they were calculated

Our web collection on statistics for biologists contains articles on many of the points above.

Software and code

Policy information about availability of computer code

Data collection	No software used.
Data analysis	Amira software version 5.5.0 (Visage Imaging GmbH, Berlin, Germany), JMP software (JMP®, Version 16 Pro; SAS Institute Inc., Cary, NC, USA).

For manuscripts utilizing custom algorithms or software that are central to the research but not yet described in published literature, software must be made available to editors and reviewers. We strongly encourage code deposition in a community repository (e.g. GitHub). See the Nature Portfolio guidelines for submitting code & software for further information.

Data

Policy information about availability of data

All manuscripts must include a data availability statement. This statement should provide the following information, where applicable:

- Accession codes, unique identifiers, or web links for publicly available datasets
- A description of any restrictions on data availability
- For clinical datasets or third party data, please ensure that the statement adheres to our policy

Supplementary Data 1 provides the source data behind the graphs presented in the manuscript. The corresponding author can provide additional data supporting this study's findings upon reasonable request.

Research involving human participants, their data, or biological material

Policy information about studies with human participants or human data. See also policy information about sex, gender (identity/presentation), and sexual orientation and race, ethnicity and racism.

Reporting on sex and gender	The sex of fetus specimens was determined through observation of the external genitalia. Seventy-two human fetus specimens (34 male and 38 female) were included in this study.
Reporting on race, ethnicity, or other socially relevant groupings	No socially relevant grouping in this study.
Population characteristics	The fetus specimens population is assumed to be representative of the unselected intrauterine population in Japan.
Recruitment	Fetus specimens were stored at the Congenital Anomaly Research Center of Kyoto University and Shimane University. Notably, most specimens were acquired under the Maternity Protection Law of Japan. The fetus specimens population is assumed to be representative of the unselected intrauterine population in Japan.<br>The specimens were collected between 1963 and 1995 according to the relevant regulations. The parents were not required to provide written informed consent at that time. Instead, parents provided verbal informed consent to deposit the specimens, and each participant's consent was documented in their medical records. All specimens were anonymized and de-identified.
Ethics oversight	This study, which used human fetus specimens, was approved by the Kyoto University Faculty and Graduate School of Medicine Ethics Committee (Approval numbers: E986, R0316, and R2224).

Note that full information on the approval of the study protocol must also be provided in the manuscript.

Field-specific reporting

Please select the one below that is the best fit for your research. If you are not sure, read the appropriate sections before making your selection.

☒ Life sciences ☐ Behavioural & social sciences ☐ Ecological, evolutionary & environmental sciences

For a reference copy of the document with all sections, see nature.com/documents/nr-reporting-summary-flat.pdf

Life sciences study design

All studies must disclose on these points even when the disclosure is negative.

Sample size	Seventy-two human fetus specimens (34 male and 38 female) were included in this study. This number of samples was determined by selecting fetal specimens with normal externals and internals from a limited number of specimen collections.
Data exclusions	No data were excluded from the analysis.
Replication	The pelvimetry measurements were taken twice for accuracy. Two authors, Toru Kanahashi and Jun Matsubayashi, have reviewed the statistical analysis results and confirmed their accuracy.
Randomization	Allocation was not random.<br>Multiple regression analysis was conducted to investigate sex differences in pelvic morphology, with CRL as a covariate to account for size variations.
Blinding	The sex of the fetal specimens was determined from gross observation of the external genitalia. Pelvimetry was conducted on a different day, using a three-dimensional reconstructed pelvis from magnetic resonance images. Sex does not affect the measurements, as it is challenging to determine sex from magnetic resonance images and three-dimensional reconstructed pelvis.

Reporting for specific materials, systems and methods

We require information from authors about some types of materials, experimental systems and methods used in many studies. Here, indicate whether each material, system or method listed is relevant to your study. If you are not sure if a list item applies to your research, read the appropriate section before selecting a response.

Materials & experimental systems

n/a	Involved in the study
☒	☐ Antibodies
☒	☐ Eukaryotic cell lines
☒	☐ Palaeontology and archaeology
☒	☐ Animals and other organisms
☒	☐ Clinical data
☒	☐ Dual use research of concern
☒	☐ Plants

Methods

n/a	Involved in the study
☒	☐ ChIP-seq
☒	☐ Flow cytometry
☒	☐ MRI-based neuroimaging

Plants

Seed stocks	*Report on the source of all seed stocks or other plant material used. If applicable, state the seed stock centre and catalogue number. If plant specimens were collected from the field, describe the collection location, date and sampling procedures.*
Novel plant genotypes	*Describe the methods by which all novel plant genotypes were produced. This includes those generated by transgenic approaches, gene editing, chemical/radiation-based mutagenesis and hybridization. For transgenic lines, describe the transformation method, the number of independent lines analyzed and the generation upon which experiments were performed. For gene-edited lines, describe the editor used, the endogenous sequence targeted for editing, the targeting guide RNA sequence (if applicable) and how the editor was applied.*
Authentication	*Describe any authentication procedures for each seed stock used or novel genotype generated. Describe any experiments used to assess the effect of a mutation and, where applicable, how potential secondary effects (e.g. second site T-DNA insertions, mosiacism, off-target gene editing) were examined.*
